# The matrix protein Fibulin-5 is at the interface of tissue stiffness and inflammation in fibrosis

**DOI:** 10.1038/ncomms9574

**Published:** 2015-10-15

**Authors:** Manando Nakasaki, Yongsung Hwang, Yun Xie, Sunny Kataria, Rupali Gund, Edries Y. Hajam, Rekha Samuel, Renu George, Debashish Danda, Paul M.J., Tomoyuki Nakamura, Zhouxin Shen, Steve Briggs, Shyni Varghese, Colin Jamora

**Affiliations:** 1 IFOM-inSTEM Joint Research Laboratory, Centre for Inflammation and Tissue Homeostasis (inStem), NCBS GKVK Post, Bellary Road, Bangalore 560065, India; 2 Department of Bioengineering, University of California, San Diego, 9500 Gilman Drive, La Jolla, California 92093-0695, USA; 3 Center for Stem Cell Research, Christian Medical College, Ida Scudder Road, Vellore, Tamil Nadu 632004, India; 4 Department of Dermatology, Venereology and Leprosy, Christian Medical College, Ida Scudder Road, Vellore, Tamil Nadu 632004, India; 5 Department of Rheumatology, Christian Medical College, Ida Scudder Road, Vellore, Tamil Nadu 632004, India; 6 Department of Surgery, Christian Medical College, Ida Scudder Road, Vellore, Tamil Nadu 632004, India; 7 Department of Pharmacology, Kansai Medical University, 10-15 Fumizono-cho, Moriguchi, Osaka 570-8506, Japan; 8 Section of Cell and Developmental Biology, Division of Biological Sciences, University of California, San Diego, 9500 Gilman Drive, La Jolla, California 92093-0380, USA

## Abstract

Fibrosis is a pervasive disease in which the excessive deposition of extracellular matrix (ECM) compromises tissue function. Although the underlying mechanisms are mostly unknown, matrix stiffness is increasingly appreciated as a contributor to fibrosis rather than merely a manifestation of the disease. Here we show that the loss of Fibulin-5, an elastic fibre component, not only decreases tissue stiffness, but also diminishes the inflammatory response and abrogates the fibrotic phenotype in a mouse model of cutaneous fibrosis. Increasing matrix stiffness raises the inflammatory response above a threshold level, independent of TGF-β, to stimulate further ECM secretion from fibroblasts and advance the progression of fibrosis. These results suggest that Fibulin-5 may be a therapeutic target to short-circuit this profibrotic feedback loop.

## Abstract (web summary)

Stiffness in the extracellular matrix is thought to contribute to pathological cutaneous fibrosis. Here, the authors identify the elastic fibre protein Fibulin-5 as a link and potential therapeutic target mediating the transition of cutaneous stiffening to fibrosis.

## Graphical abstract

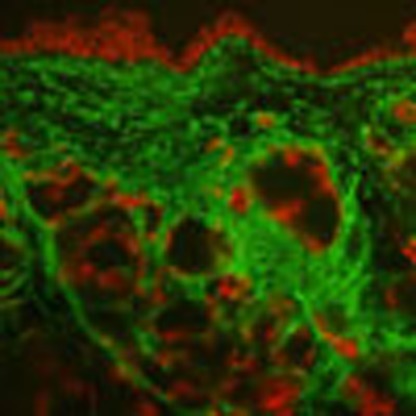

A hallmark of fibrosis is the secretion of excessive fibrous connective tissue, which is associated with a multitude of diseases leading to organ failure^1^. The critical event in the development of fibrosis is the aberrant and sustained activation of fibroblasts into myofibroblasts^2^. Myofibroblasts produce elevated levels of extracellular matrix (ECM) and secreted factors, which provides both mechanical and biochemical cues that influence the function of neighbouring cells. The formation of a dense fibrous tissue in the affected area increases the local tissue stiffness^3^. Fibroblast activation is part of the normal response to tissue damage and its activity is immediately terminated on completion of repair. However, in progressive fibrotic pathology, myofibroblasts remain activated and persistently contribute to excessive ECM deposition. Research *in vitro* suggests a connection between matrix stiffness and fibrogenesis^4^,^5^,^6^, but the mechanisms by which matrix mechanical properties contribute to fibrosis is an open question.

To probe the functional contribution of tissue stiffness to fibrosis, we used a transgenic mouse, which expresses the transcription factor Snail in the epidermis of the skin and exhibits features of fibrotic skin^7^. The Snail transgenic (Snail Tg) skin expresses elevated levels of Fibulin-5, an ECM protein involved in the formation of elastic fibres. Interestingly, the loss of Fibulin-5 was sufficient to decrease tissue stiffness, attenuate fibroblast activation, and avert the fibrotic changes in the skin. We further demonstrate that the abrogation of the fibrotic phenotype is accomplished through the dampening of the inflammatory response, which is an established prerequisite for fibrosis progression^8^. Our findings reveal how dysregulation of the ECM, independent of TGF-β signalling, provides a favourable microenvironment for fibrogenesis wherein reciprocal exchange of signals between fibroblasts and immune cells sustain fibroblast activation that fuels fibrogenesis.

## Results

### Stiff skin in neonatal Snail Tg mice

**Figure 1:**
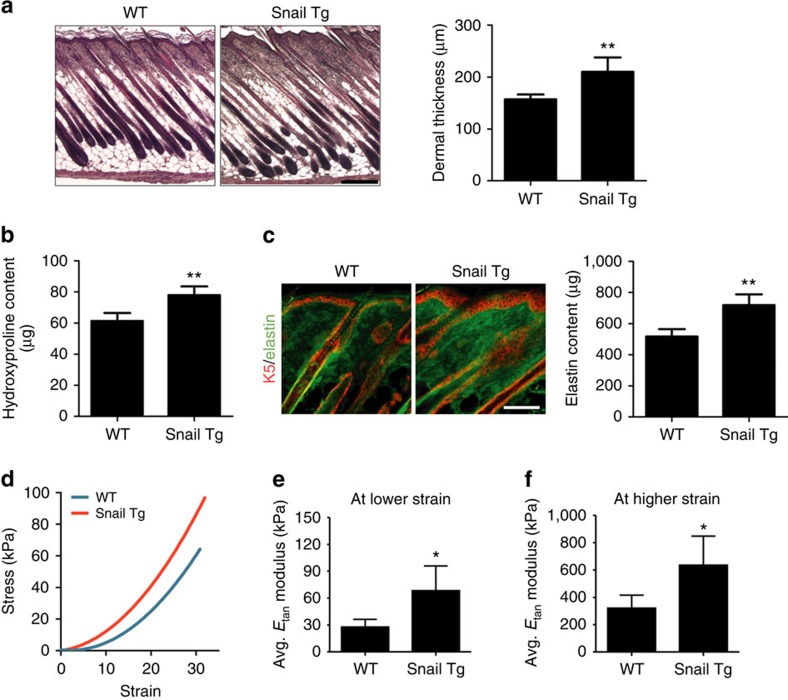
The Snail transgenic skin exhibits increased tissue stiffness. (**a**) Left: haematoxylin and eosin-stained P9 skin sections. Scale bar, 200 μm. Right: quantitation of dermal thickness. (**b**) Content of hydroxyproline extracted from the P9 whole skin of WT or Snail Tg mice. (**c**) WT (left) and Snail transgenic (Snail Tg; right) skin sections from P9 mice were stained for elastin (green) and keratin 5 (K5; red). Scale bar, 100 μm. The right graph shows the quantitation of elastin content in P9 whole skin. (**d**) Stress–strain curves for P9 WT (blue) and Snail Tg (red) skin. Average tangent modulus of P9 WT and Snail Tg skin at (**e**) lower strain (0 to 10% strain) and (**f**) higher strain (20 to 30% strain). All data represent mean±s.d. of at least six samples. *P<0.05, and **P<0.01 as compared with WT. (**a**–**c**) Statistical analyses were performed by the unpaired Mann–Whitney *U*-test. (**e**,**f**) Statistical analyses were performed with Student's *t*-test.

The Snail transgenic mouse exhibits a significant increase in the thickness of the dermal compartment of the skin as early as postnatal day 9 (Fig. 1a). This increase in dermal thickness was accompanied by excessive accumulation of the major ECM components, collagen and elastin (Fig. 1b,c). The amount of elastic fibres and collagen became progressively more pronounced in the adult transgenic skin (Supplementary Fig. 1a,b). We also examined whether the early signs of the increase in the dermal contents of ECM proteins can increase tissue stiffness, which would reduce the compliance of organs and ultimately compromise its function. The stiffness of the skin tissue was determined using stress–strain measurements. The average tangent modulus quantified for both low (elastin) and high (collagen) strains^9^,^10^,^11^ were found to be significantly higher for Snail Tg compared with wild-type (WT) skin (Fig. 1d,f).

### Loss of Fibulin-5 attenuates the fibrotic phenotype

**Figure 2:**
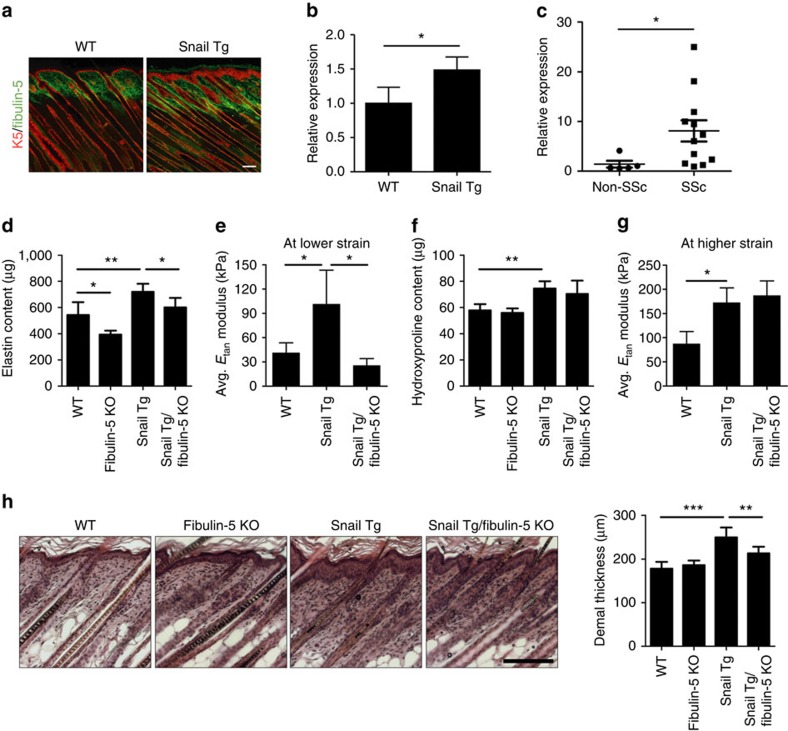
Fibulin-5-mediated elastic fibre accumulation contributes to tissue stiffness. (**a**) Skin sections from P9 mice were subjected to immunofluorescence for Fibulin-5 (green) and keratin 5 (K5; red). Scale bar, 100 μm. (**b**) Fibulin-5 protein levels following normalization with tubulin levels (Supplementary Fig. 1b). (**c**) Fibulin-5 gene expression in human systemic sclerosis skin (SSc, *n*=12) and healthy skin (non-SSc, *n*=5). Data represent mean±s.e.m. *P<0.05. (**d**) Content of elastin extracted from the P9 whole skin. (**e**) Average tangent modulus of P9 skin at lower strain (0 to 10% strain). (**f**) Content of hydroxyproline extracted from P9 whole skin. (**g**) Average tangent modulus of P9 skin at higher strain (20 to 30% strain). (**h**) Haematoxylin and eosin-stained P9 skin sections. Scale bar, 100 μm. The right bar graph shows the quantitation of dermal thickness. Unless otherwise noted, all data represent mean±s.d. of at least six samples, where *P<0.05, **P<0.01 and ***P<0.001. (**c**,**d**,**f**,**h**) Statistical analyses were performed by the unpaired Mann–Whitney *U*-test. (**b**,**e**,**g**) Statistical analyses were performed with Student's *t*-test.

It is well-known that excessive deposition of collagen fibres promotes fibrosis formation by providing the substrate for fibroblast adhesion and increasing local tissue stiffness^5^. However, comparatively little is known about the contribution of elastic fibres to fibrosis although elevated elastic fibre levels have been reported in fibrotic tissues^12^,^13^. Fibuin-5 is a key regulator of elastic fibre assembly and it is elevated in the postnatal day 9 Snail Tg skin (Fig. 2a,b and Supplementary Fig. 1c). This increase in Fibulin-5 expression is maintained in the adult mouse (Supplementary Fig. 1d), and correlates with the increased amount of elastic fibres (Supplementary Fig. 1a). Interestingly, Fibulin-5 RNA and protein levels are also increased in the skin of patients with systemic scleroderma, a progressive autoimmune disease in which fibrosis is a defining feature (Fig. 2c and Supplementary Fig. 1f). To test whether the increased elastic fibres in the Snail Tg skin contributes to fibrogenesis, we modulated the amount of elastic fibres in the Snail Tg mouse by crossing it with the Fibulin-5 null mouse^14^,^15^. The skin of the Snail Tg mouse had ∼30% more elastin relative to the WT skin and removal of Fibulin-5 restored elastic fibre levels to near WT levels (Fig. 2d and Supplementary Fig. 1e), without affecting collagen content (Fig. 2f). Likewise, removal of Fibulin-5 from the skin of the Snail Tg mouse resulted in a reduction in the tangent modulus corresponding to low strain (elastin) while no such decrease was observed for the tangent modulus corresponding to high strain (collagen) (Fig. 2e,g). The decrease in elastic fibre content in the skin from the Snail Tg/Fibulin-5 knockout (KO) mouse was also reflected in a significant decrease in the dermal thickness in the skin (Fig. 2h). Therefore, this system allowed us to assess the specific contribution of elastic fibres to the process of fibrogenesis.

**Figure 3:**
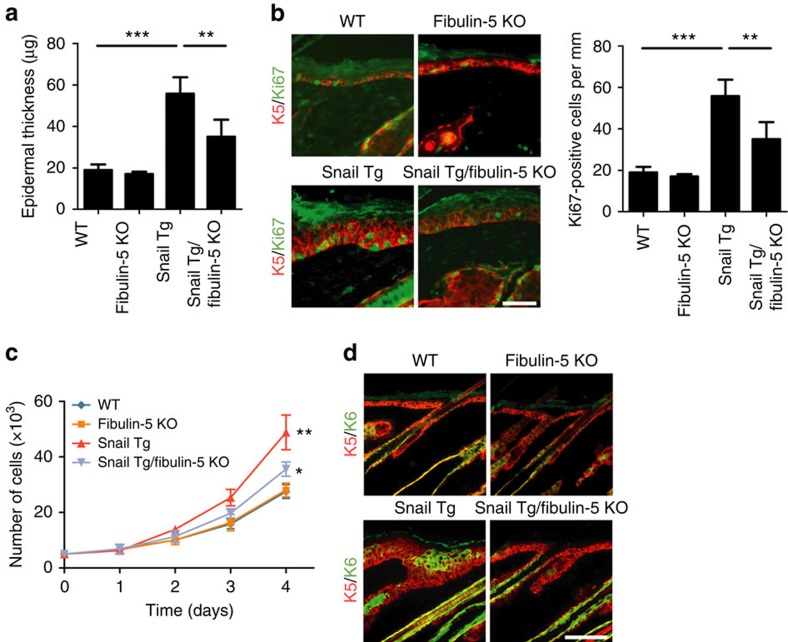
Loss of Fibulin-5 attenuated epidermal hyperplasia in the Snail Tg skin. (**a**) Quantitation of epidermal thickness, which was determined in Fig. 2h. (**b**) P9 skin sections stained for Ki-67 (green) and Keratin 5 (K5; red). Scale bar, 50 μm. Corresponding quantitation of Ki-67-positive cells is shown in the right. (**a**,**b**) Data represent mean±s.d. of at least six samples. **P<0.01. Statistical analyses were performed by the unpaired Mann–Whitney *U*-test. (**c**) Proliferation of primary mouse keratinocytes grown in media conditioned with dermis isolated from WT, Fibulin-5 KO, Snail Tg or Snail Tg/Fibulin-5 KO mouse skin. **P<0.01 as compared with WT. *P<0.05 as compared with Snail Tg. Statistical analyses were performed with Student's *t*-test. (**d**) P9 skin sections stained for Keratin 6 (K6, green) and Keratin 5 (K5: red). Scale bar, 50 μm.

To characterize the consequence of reducing the amount of elastic fibres to WT levels in the skin of Snail Tg mouse, we examined its effect on various features of fibrotic tissues such as epidermal hyperplasia^16^,^17^. Interestingly, the skin of the Snail Tg/Fibulin-5 KO mouse exhibited a decreased epidermal thickness compared with the hyperplastic Snail Tg epidermis^7^ (Fig. 3a). The loss of the epidermal hyperplasia can be at least partially attributed to the decreased amount of proliferating keratinocytes in the basal layer of the epidermis (Fig. 3b). The effect of Fibulin-5 located in the dermal compartment of the skin coincides with our previous finding that the signals promoting epidermal keratinocyte proliferation in the skin of the Snail Tg mouse emanate from the dermis^7^. We therefore examined the proliferative effect of secreted factors from the dermal tissue with or without Fibulin-5. Conditioned media from Snail Tg dermis significantly induced keratinocyte proliferation and this hyper-proliferative effect was attenuated in the media conditioned with Snail Tg/Fibulin-5 KO dermis (Fig. 3c). These results suggest that the loss of Fibulin-5 from the skin of the Snail Tg mouse elicits a change in the dermis that inhibits its ability to promote epidermal proliferation. Concurrent with the epidermal hyperplasia in the skin of the Snail Tg mouse was the expression of keratin 6, a marker associated with keratinocytes subjected to stress (Fig. 3d). The removal of Fibulin-5 from the Snail transgenic skin likewise attenuated the epidermal keratin 6 expression, suggesting that the tissue was restored to normal homeostatic conditions.

### Fibulin-5 affects cutaneous inflammation

**Figure 4:**
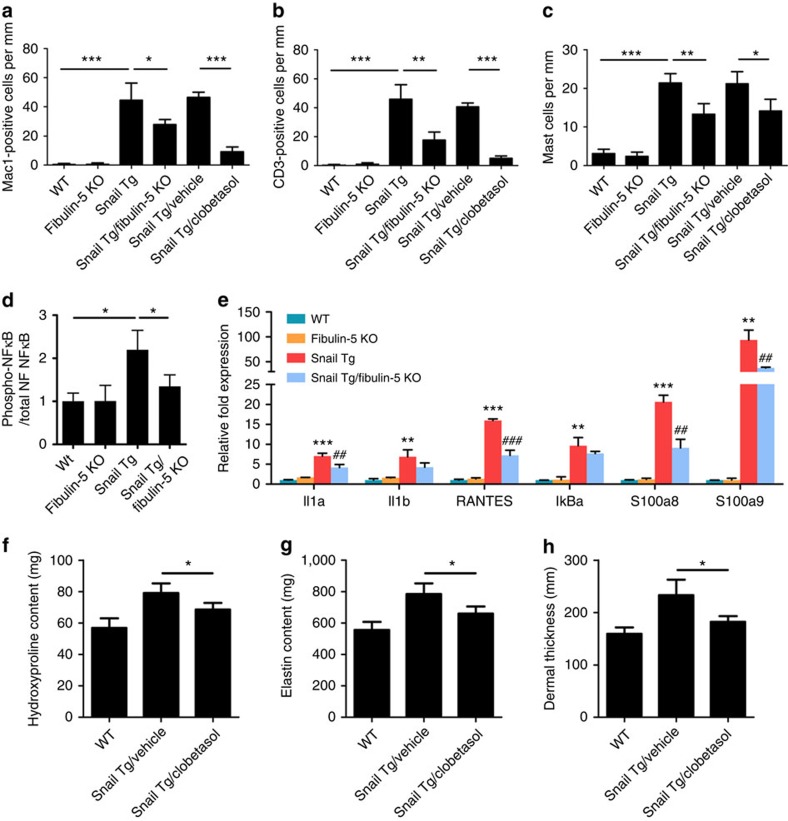
Loss of Fibulin-5 diminishes the inflammatory response in the Snail Tg skin. Quantitation of the number of infiltrated immune cells from P9 skin immunofluorescence (Supplementary Fig. 2a,c and Supplementary Fig. 3a,c). (**a**) Macrophages, (**b**) T cells and (**c**) mast cells. Snail Tg skin was treated with vehicle control (Snail Tg/vehicle) or clobetasol (Snail Tg/clobetasol). (**d**) Quantitative analysis of relative phospho-NFκB levels in P9 skin, which are estimated from the band intensities in western blotting (Supplementary Fig. 2d) were normalized by those for total NFκB. (**e**) Quantitative PCR of pro-inflammatory NFκB target genes in the whole skin of P9 mice. Data represent mean±s.d. of six biological replicates assayed in triplicate. **P<0.01 and ***P<0.001 as compared with WT. ^##^*P*<0.01 and ^###^*P*<0.001 as compared with Snail Tg. Extracts from the P9 whole skin from WT and Snail Tg treated either with control vehicle or clobetasol were probed for (**f**) hydroxyproline or (**g**) elastin content. (**h**) Quantitation of epidermal thickness from haematoxylin and eosin-stained P9 skin sections (Supplementary Fig. 3d). Unless otherwise noted, all data represent mean±s.d. of at least six samples, where *P<0.05, **P<0.01 and ***P<0.001. (**a**–**c** and **f**–**h**) Statistical analyses were performed by the unpaired Mann–Whitney *U*-test. (**d**,**e**) Statistical analyses were performed with Student's *t*-test.

We then investigated the mechanism by which the loss of Fibulin-5 and resulting decrease in elastin content can impact the proliferation of epidermal keratinocytes. Epidermal hyperplasia is often associated with local inflammation^7^,^17^, which is also a critical component of fibrogenesis^8^. We therefore tested whether Fibulin-5 can impact epidermal hyperplasia by modulating the inflammatory response in the Snail transgenic skin. The dermis in the Snail Tg skin showed elevated levels of macrophages, T cells and mast cells (Fig. 4a–c), but they were significantly decreased in the Snail Tg/Fibulin-5 KO dermis (Fig. 4a–c and Supplementary Fig. 2a–c). Moreover the level of active NFκB (phosphorylated at S276), which is a key regulator of pro-inflammatory genes, was dependent on the expression of Fibulin-5 (Fig. 4d and Supplementary Fig. 2d,e). Consistent with this observation, the expression levels of known NFκB target genes that mediate inflammation are higher in Snail Tg skin compared with WT skin and lower in the Snail Tg/Fibulin-5 KO skin (Fig. 4e and Supplementary Fig. 2f). These data indicate that the ECM associated protein, Fibulin-5, has a significant impact on establishing an inflammatory microenvironment found in the Snail transgenic skin.

### Dependence of fibrosis on inflammation in the Snail Tg mouse

Since the depletion of Fibulin-5 lowers the stiffness of the skin of the Snail Tg mouse, we tested the potential role of tissue stiffness in establishing a robust inflammatory microenvironment. Inflammation is an indispensable component in the initiation of fibrosis, but thus far tissue stiffness is primarily viewed as a consequence, and not a major cause of this pathological condition^8^. To first establish whether the inflammatory response observed in the Snail Tg skin contributes to the cutaneous fibrosis, we treated the skin of the Snail Tg mouse with clobetasol propionate, a commonly used anti-inflammatory regimen^18^, and this caused a significant reduction in the infiltration of immune cells (Fig. 4a–c and Supplementary Fig. 3a–c). Consistent with our previous results linking inflammation and epidermal hyperplasia^7^, the thickness of the Snail transgenic epidermis was reduced on the clobetasol treatment (Supplementary Fig. 3d). Interestingly, collagen (Fig. 4f), elastin (Fig. 4g) and Fibulin-5 (Supplementary Fig. 3e) contents were also significantly reduced after immunosuppression. The thickness of the dermal compartment of the skin was likewise reduced when inflammation was decreased (Fig. 4h). These results illustrate the positive feedback loop that exists between the inflammatory response and ECM proteins that coordinately elevates both inflammation and tissue stiffness to a critical level that facilitates fibrogenesis.

### The inflammation—Fibulin-5 axis impacts fibroblast activation

**Figure 5:**
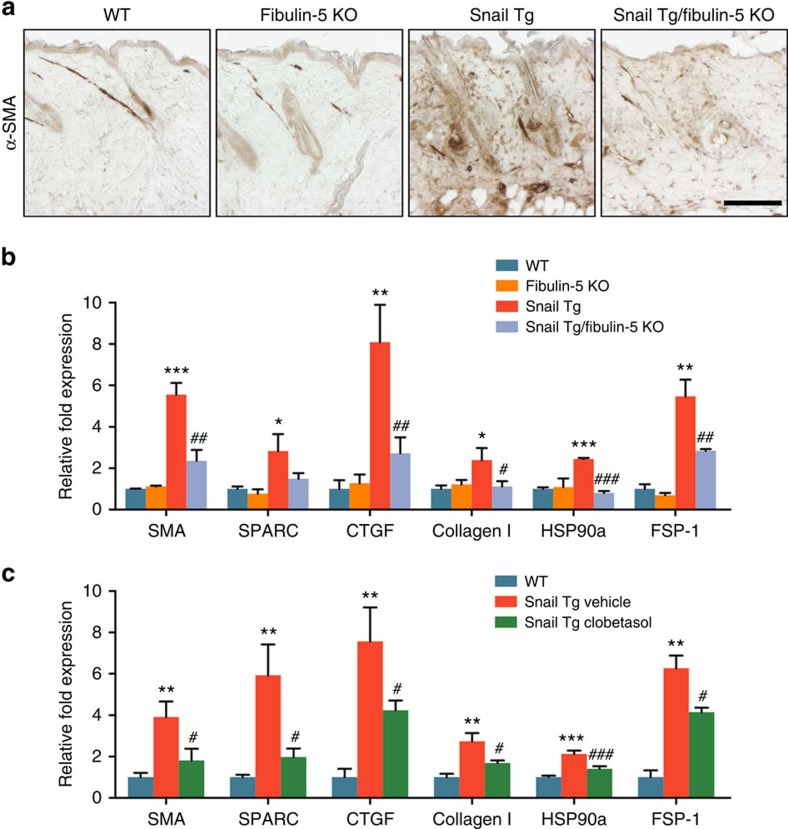
Loss of Fibulin-5 diminished fibroblast activation in the fibrotic Snail Tg skin. (**a**) Immunohistochemistry of skin sections from 8-week-old mice with antibodies recognizing α-smooth muscle action (SMA), a marker for activated fibroblasts. Scale bar, 100 μm. (**b**) Quantitative PCR (qPCR) of fibrosis-related genes in the whole skin of P9 mice. Data represent mean±s.d. of three samples. *P<0.05, **P<0.01 and ***P<0.001 as compared with WT. ^#^*P*<0.05, ^##^*P*<0.01 and ^###^*P*<0.001 as compared with Snail Tg. (**c**) qPCR of fibrosis-related genes in the P9 whole skin isolated from WT (blue), vehicle-treated Snail Tg (red) and clobetasol-treated Snail Tg mice. Data represent mean±s.d. of six biological replicates assayed in triplicate. **P<0.01 and ***P<0.001 as compared with WT. ^#^*P*<0.05 and ^###^*P*<0.001 as compared with Snail Tg treated with vehicle. Statistical analyses were performed with Student's *t*-test.

Given the effect of the attenuated inflammatory response in the Snail Tg/Fibulin-5 KO on the ECM content of the skin, we tested whether other biochemical hallmarks of fibrosis were affected. Immunohistochemical analysis reveals that the number of cells positive for smooth muscle actin (SMA), and other markers of activated fibroblasts, was significantly elevated in the skin of the Snail Tg mouse and decreased in the absence of Fibulin-5 (Fig. 5a,b and Supplementary Fig. 4a,b). If the effect of diminishing fibrosis in the Snail transgenic mouse lacking Fibulin-5 occurred through the loss of inflammation, we hypothesized that an anti-inflammatory regimen would phenocopy the genetic ablation of Fibulin-5. Similar to the effect of removing Fibulin-5, the skin of the Snail Tg mouse treated with clobetasol showed decreased expression levels of fibroblast activation-related genes compared with the vehicle control (Fig. 5c). These observations suggest that Fibulin-5 depletion attenuated fibrosis formation partly through the diminished activity of dermal fibroblasts in the skin of the Snail Tg mouse.

### Matrix stiffness potentiates chemokine gene expression

**Figure 6:**
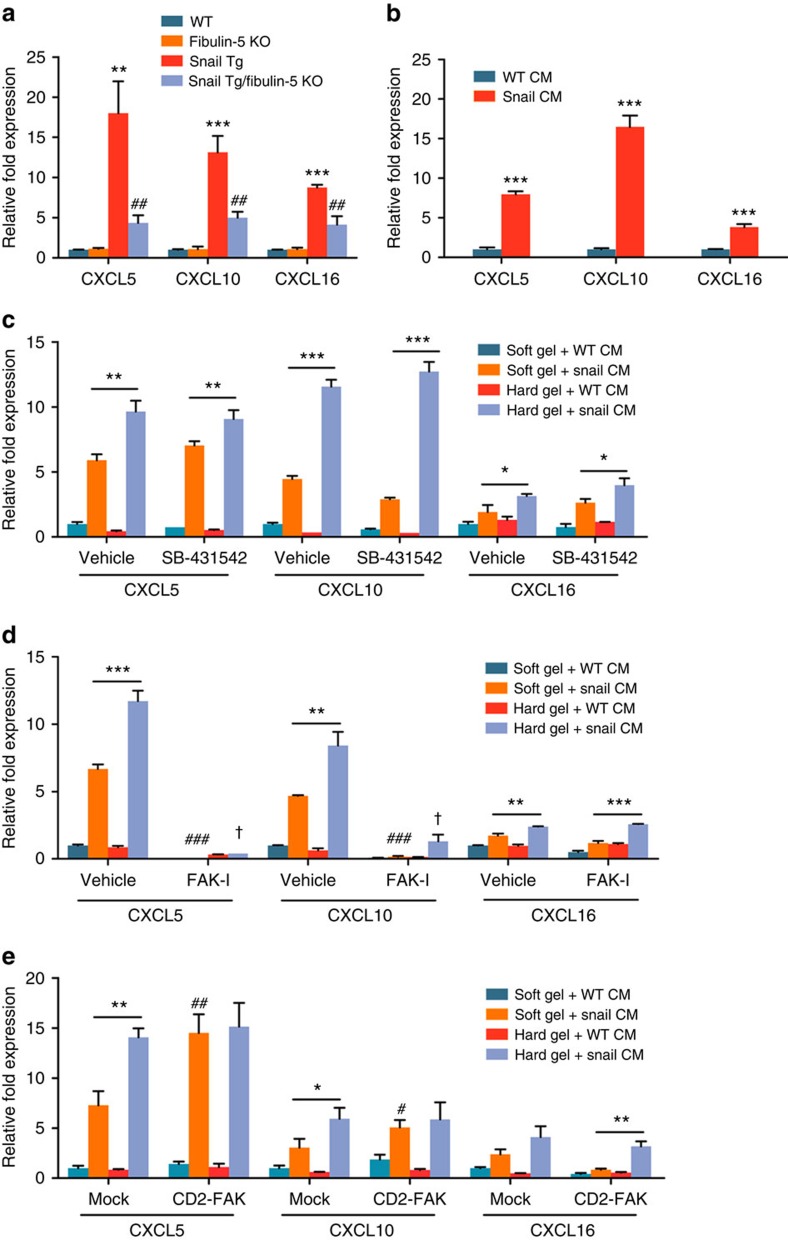
Chemokine gene expression with varying stiffness. (**a**) Quantitative PCR (qPCR) of chemokine genes in the whole skin of P9 mice. Data represent mean±s.d. of six biological replicates assayed in triplicate. **P<0.01 and ***P<0.001 as compared with WT. ^##^*P*<0.01 as compared with Snail Tg. (**b**) Cultured dermal fibroblasts were treated with conditioned media from Snail Tg keratinocytes (Snail CM) or WT keratinocytes (WT CM) for 18 h. Chemokine gene expression of treated fibroblasts was determined by qPCR. ***P<0.001 as compared with WT CM. (**c**) Dermal fibroblasts were plated on collagen-coated polyacrylamide hydrogels of varying stiffness (soft (2 kPa) and hard (5 kPa) gel). Cells were incubated with WT CM or Snail CM for 18 h with or without the presence of TGF-β receptor inhibitor SB-431542 at the concentration of 10 μM. Chemokine gene expression in treated fibroblasts was determined by qPCR. (**d**) Dermal fibroblasts were treated as in **c** with or without the presence of FAK inhibitor, PF 573228, at the concentration of 20 nM (FAK-I). Chemokine gene expression was determined by qPCR. (**e**) Dermal fibroblasts were transfected with a mock or CD2-FAK vector were treated as in **d**. Chemokine gene expression was determined by qPCR. Unless otherwise noted, data represent mean±s.d. of six samples, where *P<0.05, **P<0.01 and ***P<0.001. (**d**) ^##^*P*<0.01, and ^###^*P*<0.001 as compared with Soft gel+Snail CM (vehicle). ^†^*P*<0.001 as compared with Hard gel+Snail CM. (**e**) ^#^*P*<0.05, and ^##^*P*<0.01 as compared with Soft gel+Snail CM (mock). (**a**–**e**) Statistical analyses were performed with Student's *t*-test.

Given our observations of the role of Fibulin-5 in recruiting macrophages, T cells and mast cells into the skin of the Snail Tg mouse (Fig. 4a–c), we focused our attention on the mechanism by which an ECM protein can influence an inflammatory response. We found several chemokine genes such as CXCL5, CXCL10 and CXCL16 that are upregulated in the skin of the Snail Tg mouse and are significantly diminished on loss of Fibulin-5 (Fig. 6a). Interestingly, although TGF-β has been regarded as a primary regulator of fibrogenesis^4^, expression of all three isoforms was not upregulated in our fibrosis model (Supplementary Fig. 5a). These results prompted us to investigate the possibility that the Fibulin-5-dependent increase in tissue stiffness is an important contributor to the generation of a robust inflammatory response. Recently, fibroblasts have been revealed to play a role as a hub of inflammation^8^,^19^ and we hypothesized that secreted factors from the Snail Tg epidermis work synergistically with matrix stiffness to elevate inflammatory cytokine expression in dermal fibroblasts to a critical threshold level conducive to fibrogenesis. We first tested whether dermal fibroblasts treated with conditioned media from Snail Tg keratinocytes can induce chemokine gene expression. Indeed, Snail conditioned media was capable of inducing the expression of the same chemokines in fibroblasts that are induced in the Snail Tg mouse *in vivo* (Fig. 6b). However, this experiment was performed on cells cultured on a plastic tissue culture plate, which has a stiffness that far exceeds what is found in normal or pathological tissues. To test whether subtle changes in tissue stiffness—as documented in both the development of fibrosis in diseased human tissues, as well as the skin of the neonatal Snail Tg mouse—could contribute to the strength of the inflammatory response mounted by dermal fibroblasts, we cultured cells on hydrogels with defined stiffness. To establish the baseline level of inflammatory gene expression, we plated fibroblasts on ‘soft' hydrogel with a stiffness of 2 kPa, which is within the range of stiffness for normal human dermis^20^,^21^. To mimic the ∼2.5-fold increase in tissue stiffness of the skin of the Snail Tg mouse (Fig. 2e), we plated cells on a ‘hard' hydrogel with a stiffness of 5 kPa. The fold induction of chemokine genes in response to Snail conditioned media was significantly higher on the stiffer hydrogel than that on the soft hydrogel (Fig. 6c). Moreover, the small change (∼2.5-fold) in matrix stiffness was sufficient to enhance the response of dermal fibroblasts to pro-inflammatory signals, leading to upregulation of chemokine gene expression levels. However this small change was insufficient to induce TGF-β gene expression (Supplementary Fig. 5b) or Smad2/3 phosphorylation, which is a downstream effect of TGF-β signalling (Supplementary Fig. 5c). Although we observed an increase in Smad2/3 phosphorylation level in the Snail Tg skin as seen in fibrotic tissues^4^, Fibulin-5 depletion only reduced this increase by ∼25% (Supplementary Fig. 5d). Importantly, a TGF-β receptor inhibitor (SB-431542) did not affect the chemokine gene induction (Fig. 6c), implying that the influence of Fibulin-5 on the production of inflammatory cytokines in dermal fibroblasts is independent of TGF-β signalling. Interestingly, the approximately twofold difference in chemokine expression levels between the soft and hard hydrogels (Fig. 6c) is comparable to the two to threefold difference in their *in vivo* expression levels between the skin of the Snail Tg mouse with or without Fibulin-5 (Fig. 6a). Instead of its effect on tissue stiffness, an alternative explanation for the ability of Fibulin-5 to reverse the fibrotic phenotype in the Snail transgenic mouse could be its direct effect on epidermal keratinocytes and/or dermal fibroblasts. However, this possibility was ruled out by the following findings: (i) dermal fibroblasts retained their ability to induce chemokine gene expression in response to conditioned media from Snail Tg keratinocytes regardless of the presence or absence of the Fibulin-5 gene (Supplementary Fig. 6a); (ii) dermal fibroblasts freshly isolated from either Snail Tg skin or Snail Tg/Fibulin-5 KO skin exhibited similar levels of response to the conditioned media from Snail Tg keratinocytes or WT fibroblasts (Supplementary Fig. 6b); and (iii) conditioned media from the Snail Tg and Snail Tg/Fibulin-5 KO epidermis induced similar levels of chemokine gene expression in fibroblasts, indicating that pro-inflammatory signals from epidermis were not affected by Fibulin-5 expression in epidermis (Supplementary Fig. 6c).

We then focused on the possible mechanism by which dermal fibroblast would sense the changes in tissue/substrate stiffness. A key pathway in mechanotransduction is integrin signaling, which can activate intracellular FAK signalling (via phosphorylation)^22^. Such signalling has been shown to be involved in inflammatory response in skin fibrosis^23^. Consistent with this, we found that FAK phosphorylation was increased in the fibrotic skin of the Snail Tg mouse and returned to WT levels on Fibulin-5 depletion (Supplementary Fig. 7a). Similar results were obtained on hydrogels where hard matrices increased phosphorylated FAK in dermal fibroblasts, while treatment with Snail Tg conditioned media did not further increase FAK phosphorylation (Supplementary Fig. 7b). We short-circuited this signalling pathway with a FAK inhibitor (Supplementary Fig. 7b) and found that it abrogated the expression of both CXCL5 and CXCL10 in fibroblasts (Fig. 6d). We also found that a constitutively active form of FAK (CD2-FAK)^24^ increased the expression of these two chemokines on soft gels to levels comparable to fibroblasts plated on hard gels (Fig. 6e and Supplementary Fig. 7c). Taken together, these results suggest that elevated tissue stiffness in the skin of the Snail Tg mouse primes dermal fibroblasts to mount a vigorous inflammatory response required to promote fibrogenesis. Interestingly, CXCL16 seemed to buck the trend of being susceptible to FAK activation (Fig. 6d,e), suggesting an alternative route of mechanotransduction that regulates the expression of this cytokine. Among the candidates responsible for this are the transcription factors YAP/TAZ that have been shown to modulate mechanotransduction *in vitro*^25^. However, we found that downstream targets of these factors are actually downregulated in our Snail model of tissue fibrosis (Supplementary Fig. 8). A similar phenomenon has been reported in a model of laminin-A-mediated increase in tissue stiffness in which the YAP protein is decreased^26^. Together these results suggest that other important pathways are awaiting identification that can mediate changes in extracellular matrix stiffness with changes in gene expression.

## Discussion

As collagen accounts for ∼75% of the ECM proteins in the skin, a large body of research has focused on collagen as a major constituent in fibrotic tissue but scant attention has been paid to the role(s) of elastic fibres, which accounts for ∼8% of cutaneous ECM^27^,^28^. Elastic fibres are abundantly deposited in fibrotic skin^29^, and our data suggests that they play two interrelated roles in the fibrosis: tissue stiffening and inflammation, and together these conspire to promote the progression of fibrosis. The loss of Fibulin-5 is known to cause impairment of elastic fibre formation without obvious alterations in the status of collagen^14^,^15^, which was consistent with our observation that Fibulin-5 depletion did not alter the elevated collagen content in the neonatal skin of the Snail Tg mouse.

Several other constituents of elastic fibres are involved in the progression of fibrosis. LTBP-4 was recently identified to promote elastic fibre assembly by interacting with Fibulin-5 (ref. 30). LTBP-4 was originally found as a latent TGF-β-binding protein that sequesters the profibrotic cytokine TGF-β regulating its availability for binding to the TGF-β receptor and its mutation has been found to cause fibrosis in muscle^31^. Another profibrotic effect was found in the mutation of integrin-binding domain of fibrillin-1, which is the main constituent of extracellular microfibrils in elastic fibres^32^,^33^. These findings imply the critical role elastic fibres play not only in conferring physical properties of the matrix but also in modulating signalling pathways relevant for fibrogenesis. Alterations in Fibulin-5 protein function have thus far not been reported to cause profibrotic effects, and our work provides the first *in vivo* evidence elastic fibre assembly can regulate important cytokine gene expression.

Published research has established that normal lung and skin tissues have elastic moduli of <2 kPa and these tissues increase their stiffness by approximately twofold in a drug-induced fibrosis model^6^,^21^. Consistent with this pathophysiologically relevant range, our data demonstrated that a 2.5-fold increase in matrix stiffness (2 to 5 kPa) was sufficient to enhance the response of dermal fibroblasts to pro-inflammatory signals, leading to an enhanced chemokine gene expression. Interestingly, this small increase in matrix stiffness documented in pathological tissues was not sufficient to induce the expression of TGF-β ligands nor augment TGF-β signal transduction through Smad2/3 phosphorylation. This was surprising given the preponderance of evidence implicating TGF-β signalling in tissue fibrosis^4^ although several TGF-β signalling independent mechanisms of fibrogenesis have been suggested^34^. Our results therefore reveal additional novel players in fibrogenesis and shed light on possible reasons why TGF-β inhibition alone has not been providing promising results in clinical trials of diseases marked by tissue fibrosis^2^.

The blockade of immune cell recruitment with clobetasol, a potent corticosteroid, completely abrogated the fibrosis programme, emphasizing the role of inflammation as an indispensable primer for fibrosis. However, inflammation is not only important in fibrogenesis, but in the maintenance of the fibrotic phenotype as a partial resolution of skin symptoms was achieved in patients with the fibrotic skin disease known as diffuse systemic sclerosis treated with dexamethasone pulse therapy^35^. Our results demonstrate that the inflammatory response can be initiated by alterations in epithelial homeostasis. Interestingly, the expression of members of the Snail family of transcription factors is a common cellular response to multiple types of cellular stress that perturb tissue homeostasis^36^,^37^,^38^,^39^. Our results demonstrate how the inflammatory response, which is activated by epithelial stress signals, is further potentiated by increasing tissue stiffness. The interdependence of inflammation and tissue stiffness further comprises a positive feedback loop wherein the inflammatory response subsequently stimulates the elevated production of ECM proteins in dermal fibroblasts. These findings aid in understanding the vicious cycle between inflammation and tissue stiffening, which has been anticipated to drive fibrogenesis. Collectively, our data imply that interventions to ameliorate tissue stiffness though ECM protein levels may halt this self-sustaining fibrotic process and Fibulin-5 involved in the stiffening of fibrotic tissue may serve as a therapeutic target for progressive fibrosis.

## Methods

### Animals

C57BL6 mice engineered to express the Snail transgene in the epidermis was previously described^40^. The *Fibulin-5* KO mouse, also on the C57BL6 background, was described in ref. 14. The age of the mice used is noted in the text and results were similar for either male or female mice. All animal work was approved and adhered to the guidelines of IACUC at the University of California, San Diego and the Institute for Stem Cell Biology and Regenerative Medicine (Bangalore, India).

### Histology and immunohistochemistry

Staining for phospho-NFκB p65 (Ser276,Cell Signaling, #3037) and α-SMA (Sigma-Aldrich, clone 1A4) was performed on 8-μm paraffin sections after antigen-retrieval. Epidermal differentiation marker K5 (C.J. Lab), the wound healing marker K6 (Covance, PRB-169P), Fibulin-5 (T.N. Lab), elastin (Millipore, clone 10B8) and Ki-67 (Vector Labs, clone MM1) were stained in 8-μm frozen sections after the tissues were fixed for 10 min in 4% paraformaldehyde. Immune cell infiltrates were stained using antibodies against MAC-1 (BD Biosciences, clone M1/70) and CD3 (eBioscience, clone 17A2). Primary antibodies were used at 1:100 dilution. For mast cell staining, paraffin sections were stained with toluidine blue. For nuclear staining, Hoechst 33342 (Calbiochem) was added at a final concentration of 1 mg ml^−1^ to the secondary antibody dilution. Secondary antibodies were used at 1:100 dilution. Immunofluorescence was detected using rhodamine-X or FITC-conjugated secondary antibodies (Jackson Immunoresearch) or expression was developed using the Vectastain ABC kit (Vector Labs) according to the manufacturer's instructions. Mouse skin isolated from P9, 8-week- or 4-month-old WT and transgenic/KO animals were either frozen in OCT (Tissue-Tek, Sakura) or fixed for 2 h in Bouin's fixative and embedded in paraffin depending on the application. Paraffin sections were prepared for histology and counterstained with haematoxylin and eosin-Y (H&E). For elastic fibre staining, paraffin sections were stained with Verhoeff–Van Gieson staining (Sigma-Aldrich). Images were acquired on an Olympus Bx51 microscope with an Olympus DP70 camera. A 40 × 1.3 UPlan FL N objective (Olympus) was used for acquisition. Epidermal and dermal thickness was measured on H&E-stained sections by ImageJ software (NIH) and averaged from three different samples of each group. The number of Ki-67 positive or infiltrated immune cells in the dermis was counted, normalized by the perimeter and averaged from three different samples of each group.

### Measurement of hydroxyproline and elastin

To determine hydroxyproline content, punch-biopsied skin samples (Ø 5 mm) were homogenized in 6 N HCl and hydrolyzed by incubation at 100 °C for 16 h. Aliquots were dried and crystals were dissolved in dH_2_O by vortexing. Both standards and samples were added chloramine-T solution (BDH) followed by vortexing and incubation at room temperature for 15 min. Samples were developed by adding freshly prepared *p*-dimethylamino benzaldehyde (DMBA, Sigma-Aldrich), homogenized and incubated at 60 °C for 20 min. The optical density of each sample was measured at 562 nm and the concentration was calculated. Elastin content of punch-biopsied skin samples (Ø 5 mm) was quantified using a Fastin elastin assay kits (Biocolor) following the manufacturer's instruction.

### Western blot

Frozen skin tissue was pulverized using liquid nitrogen, dissolved in Laemmli buffer and RIPA buffer (1% Triton X-100 in PBS with 10 mM EDTA, 150 mN NaCl, 1% sodium deoxycholate and 0.1% SDS) and sonicated. Samples were resolved by SDS–PAGE and probed by immunoblotting. The band intensities in the western blots were determined by ImageJ software. Immunoblotting was performed with antibodies against phospho-NFκB p65, NFκB (Cell Signaling, #3034), α-SMA, Fibulin-5, Smad2/3 (Cell Signaling, #5678), phospho-Smad2 (Ser465/467)/Smad3 (Ser423/425) (Cell Signaling, #8823), FAK (Cell Signaling, #3285), phospho-FAK (Tyr 397, Cell Signaling, #3283) and tubulin (Sigma-Aldrich, clone B-5-1-2). Primary antibodies were used at 1:1,000 dilution and horse radish peroxidase-conjugated secondary antibodies (Jackson Immunoresearch) were used at 1:10,000 dilution.

### Skin stress–strain test

The dorsal skin of both WT and transgenic mice were isolated and cut into dog bone-shaped specimens using a scalpel. The thickness of the specimens was measured after carefully removing the underlying adipose tissue. Arrays of dots, which have a uniform distance of 1 mm with nine rows and four columns, were put on the surface of the specimen between the shoulders. Tensile tests were carried out using a custom-made testing device equipped with a force transducer (Pasco Structure System, PS-2201). The specimens were clamped using anti-slip grips and uniaxial tension was applied with a strain rate of 10 mm min^−1^. The tensile load was measured with 5 Newton (5N) of load cell and the displacement of the specimens was recorded with a high resolution digital camera. The distance between arrays of dots on the specimens, while the specimens underwent deformation, was calculated to obtain the displacement of the specimens. The average tangent modulus was calculated at 0 to 10% strain (lower strain) and at 20–30% strain in the stress–strain curve. All measurements were performed as quadruplicates for each experiment group.

### Human systemic scleroderma skin

Skin punch biopsies were taken from the arms of patients diagnosed with diffuse systemic sclerosis and non-systemic sclerosis. RNA was isolated from skin samples and subjected to Fibulin-5 gene expression analysis by quantitative PCR and immunohistochemistry. Characteristics of patient samples are displayed in Supplementary Table 1. Acquisition and processing of the tissue followed the protocol approved by the Institutional Review Board (IRB) of Christian Medical College, Vellore, India (Research & Ethics Committee). Informed consent was acquired from all patients for skin sample collection and experimentation.

### Conditioned media from dermal and epidermal tissue

Dermis isolated by incubating with dispase (Roche) from each genotype was incubated in serum-free keratinocyte media for overnight. Conditioned media were diluted 1:5 with keratinocytes media, added with 2% foetal bovine serum (FBS) and applied to a well containing 5,000 keratinocytes inoculated in a 96-well plate. Cell numbers were measured by Cell TiTer 96 Aqueous One Solution (Promega) as suggested by the manufacturer. Epidermis isolated by incubating with dispase from each genotype was incubated in serum-free DMEM for overnight. Conditioned media were used to induce chemokine gene expression in fibroblasts on hydrogels.

### Reverse Transcription Polymerase Chain Reaction (RT-PCR)

Total RNA was extracted from whole skin of each genotype mouse at P9 or fibroblasts culture using Trizol reagent (Invitrogen) and RNeasy kit (Qiagen) according to manufacturer's instructions. Complementary DNA (cDNA) was synthesized by iScript cDNA Synthesis Kit (Bio-Rad). Real-time PCR analysis was performed with primers shown in Table 1 using a CFX96 Touch Real-Time PCR Detection System (Bio-Rad). Reactions were performed using SsoFast qPCR Supermixes with EvaGreen (Bio-Rad) and experiments were carried out in triplicate from cDNA isolated from three different animals. Gene expression was normalized to actin expression levels as reference.

### Clobetasol treatment

Clobetasol (Sigma-Aldrich) 0.05% in propylene glycol/ethanol (7:3 v/v) vehicle was applied to the animal's back skin every other day from the age of day 1 to day 9. Another group of littermates received vehicle alone at the same frequency.

### Cell culture

For dermal fibroblast isolation, dermis was separated from the skin of newborn mice in 1 mg ml^−1^ of dispase for 2 h at 37 °C and digested in 2.5 mg ml^−1^ of collagenase IV (Gibco) for 2 h at 37 °C. Isolated primary dermal fibroblasts were maintained in Dulbecco's Modified Eagle Medium (DMEM) (Gibco) supplemented with 10% FBS in 5% CO_2_ at 37 °C. Primary keratinocytes were cultured in DMEM/Ham's F-12 Nutrient Medium (3:1 mix) without calcium (Gibco Invitrogen, special order custom powder media, Cat. #90- 5010EA) supplemented with 10% calcium-chelated FBS^41^. For chemokine gene induction in fibroblasts, keratinocytes isolated from WT or Snail Tg mice were incubated with serum-free keratinocyte media overnight. Dermal fibroblasts were seeded at a density of 4 × 10^4^ cell per cm^2^ on hydrogels with the stiffness of 2 and 5 kPa and stimulated with keratinocyte-conditioned media, which were diluted 1:5 in fibroblast culture media (DMEM with 10%FBS) for 18 h. FAK inhibitor PF 573228 and TGF-β receptor inhibitor SB-431542 were purchased from Sigma-Aldrich. A pLV-neo-CD2-FAK (gift from B. Weinberg, Addgene plasmid # 37013)^42^ or mock vector was electroporated into mouse dermal fibroblasts using a Nucleofector 2b device and Nucleofector kit for primary fibroblasts (Lonza).

### Hydrogel preparation

Coverslips (22 × 22 mm, Fischer Scientific) were treated with 2.5 M NaOH and a 2% aqueous solution of 3-(Trimethoxysilyl)propyl methacrylate (Sigma-Aldrich) sequentially. Solutions containing 0.1% ammonium persulfate, 0.1% tetramethylethylenediamine and variable ratios of acrylamide/bisacrylamide (Calbiochem) were delivered on the treated coverslips according to the concentration chart reported in ref. 43. Soft (2 kPa) and hard (5 kPa) gels were made based on the fact that the reported modulus of the human dermis can range from 0.1 to 10 kPa (refs 20, 21). After polymerization, the gel surface was derivatized with heterobifunctional cross-linker Sulfo-SANPAH (Thermo Fisher Scientific) and UV irradiation^44^. Rat-tail collagen (BD Biosciences) diluted in PBS at 100 μg ml^−1^ was delivered on each coverslip and incubated for 18 h at 37 °C. The hydrogels on coverslips were rinsed in PBS and UV-sterilized before cell seeding.

### Statistical analysis

Results are expressed as mean±s.d. Differences between groups were analysed by the unpaired Mann–Whitney *U*-test or Student's *t*-test using the Prism statistical program (GraphPad Software, Inc.). *P* values <0.05 were considered significant.

## Additional information

**How to cite this article:** Nakasaki, M. *et al*. The matrix protein Fibulin-5 is at the interface of tissue stiffness and inflammation in fibrosis. *Nat. Commun.* 6:8574 doi: 10.1038/ncomms9574 (2015).

## Supplementary Material

Supplementary Information Supplementary Figures 1-8 and Supplementary Table 1.

## Figures and Tables

**Table 1:** Primer sequences.

Gene	5′ primer sequence	3′ primer sequence
*β-actin*	GGGCTATGCTCTCCCTCAC	GATGTCACGCACGATTTCC
*IL-1α*	CTCTAGAGCACCATGCTACAGAC	TGGAATCCAGGGGAAACACTG
*IL-1β*	GCAACTGTTCCTGAACTCAACT	ATCTTTTGGGGTCCGTCAACT
*RANTES*	CCTCACCATCATCCTCACTGCA	TCTTCTCTGGGTTGGCACACAC
*IκBα*	GGAGCGCTTGGTGGACGATC	GCCCTGCTCACAGGCAAGAT
*S100a8*	AAATCACCATGCCCTCTACAAG	CCCACTTTTATCACCATCGCAA
*S100a9*	ATACTCTAGGAAGGAAGGACACC	TCCATGATGTCATTTATGAGGGC
*α-SMA*	ATCGTCCACCGCAAATGC	AAGGAACTGGAGGCGCTG
*SPARC*	GCTGTGTTGGAAACGGAGTTG	CTTGCCATGTGGGTTCTGACT
*CTGF*	GTGCCAGAACGCACACTG	CCCCGGTTACACTCCAAA
*col1a1*	GCCAAGAAGACATCCCTGAAG	TCATTGCATTGCACGTCATC
*HSP90a*	CAGTGATGATGAGGCTGAAG	CAGGATTTCTCGTCCAAATCG
*FSP-1*	CAGCACTTCCTCTCTCTTGG	TTTGTGGAAGGTGGACACAA
*CXCL5*	AGCTCGCCATTCATGCGGAT	CACTGCGAGTGCATTCCGCT
*CXCL10*	GTCATTTTCTGCCTCATCCTGCT	GGATTCAGACATCTCTGCTCATCA
*CXCL16*	CCTTGTCTCTTGCGTTCTTCC	TCCAAAGTACCCTGCGGTATC
*NLRP3*	ATTACCCGCCCGAGAAAGG	TCGCAGCAAAGATCCACACAG
*CRP*	ATGGAGAAGCTACTCTGGTGC	ACACACAGTAAAGGTGTTCAGTG
*p53*	CTCTCCCCCGCAAAAGAAAAA	CGGAACATCTCGAAGCGTTTA
*IL-6*	CGTGGAAATGAGAAAAGAGTTGTG	CCAGTTTGGTAGCATCCATCATTTCT
*col3a1*	CTGTAACATGGAAACTGGGGAAA	CCATAGCTGAACTGAAAACCACC
*col6a2*	AAGGCCCCATTGGATTCCC	CTCCCTTCCGACCATCCGAT
*TGF-β1*	CTCCCGTGGCTTCTAGTGC	GCCTTAGTTTGGACAGGATCTG
*TGF-β2*	CTTCGACGTGACAGACGCT	GCAGGGGCAGTGTAAACTTATT
*TGF-β3*	CCTGGCCCTGCTGAACTTG	TTGATGTGGCCGAAGTCCAAC
*ANLN*	GTTAAAACTCGAATGCAAAGGCT	AGTTGGCACTGGTGCAAAGTA
*DIAPH3*	GAGAAGCGACCCAAGTTGCAT	GAAGGGGAGGTCTCTCTTTCTT
*Fibulin-5(human)*	CTACTCGAACCCCTACTCGAC	TCGTGGGATAGTTTGGAGCTG
