# Supplementary material for: The matrix protein Fibulin-5 is at the interface of tissue stiffness and inflammation in fibrosis
Source: Nat Commun. 2015 Oct 15;6:8574. doi: 10.1038/ncomms9574 (PMC4634219; doi:10.1038/ncomms9574)
Supplement: Supplementary Information — Supplementary Figures 1-8 and Supplementary Table 1. [file ncomms9574-s1.pdf]

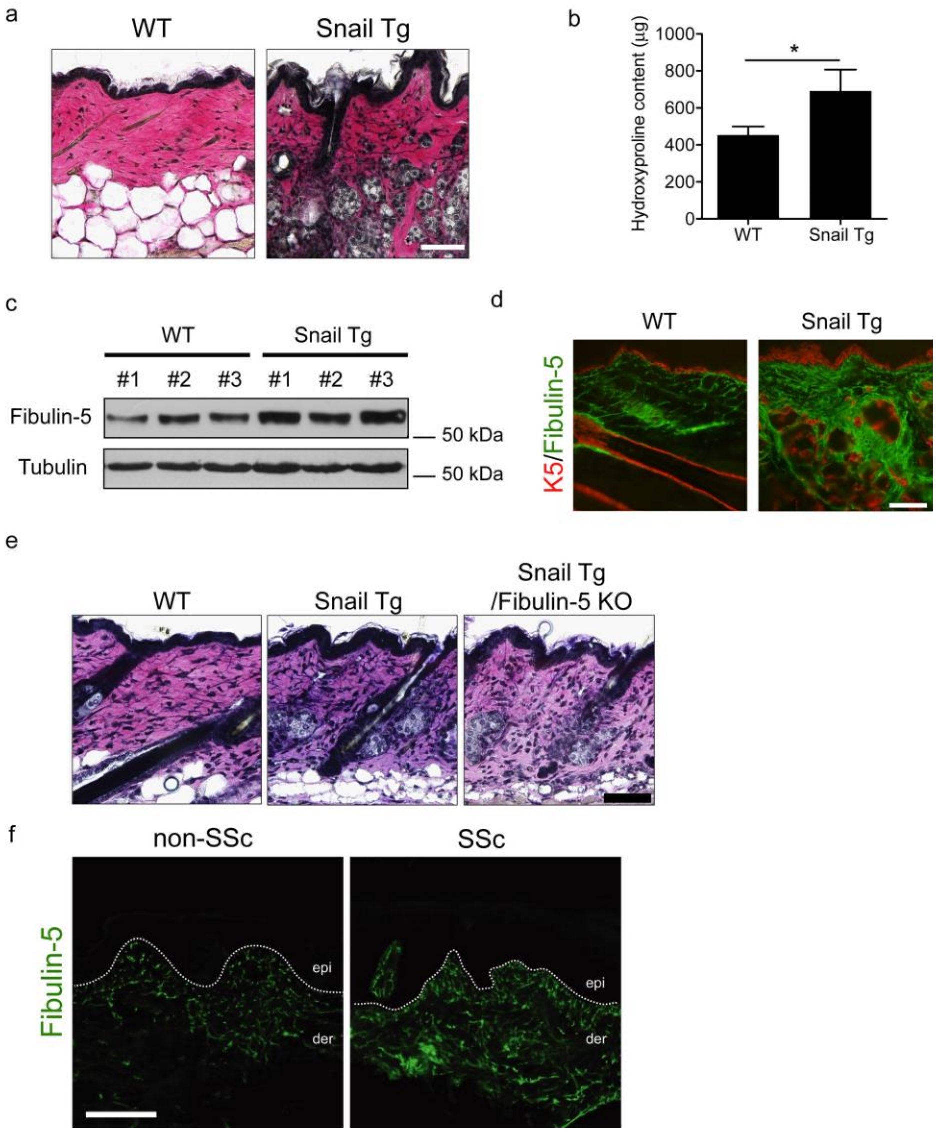

Supplementary Figure 1 Increased elastic fibers and protein expression of fibulin-5 in fibrotic Snail Tg skin. (a) Verhoeff-Van Gieson staining of 4-month-old WT and Snail Tg skin. Elastic fibers are stained in blue to black. Bar, 100 μm. (b) Collagen from the whole skin of 4 month WT or Snail Tg mice determined by hydroxyproline content. Data represent mean ± SD of four samples. $^{*}P < 0.05$. Statistical analyses were performed by the unpaired Mann-Whitney *U* test. (c) Protein extracts were prepared from whole skin from P9 WT and Snail Tg mice. Equal

amounts of proteins were resolved by SDS-PAGE and subjected to Western blotting using either an anti-fibulin-5 or an anti-tubulin as a control. (d) Skin sections from 4-month-old mice were subjected to immunofluorescence with antibodies recognizing fibulin-5 (green) and keratin 5 (K5; red), a marker of epidermal basal layer. Bar, 100 μm. (e) 8-week-old skin sections from WT, Snail Tg and Snail Tg/Fibulin-5 KO were subjected to Verhoeff-Van Gieson staining. Elastic fibers are stained in blue to black. Bar, 100 μm. (f) Skin sections from human systemic sclerosis skin (SSc) and healthy skin (non-SSc) were subjected to immunofluorescence with antibodies recognizing fibulin-5 (green) Bar, 100 μm. epi: epidermis, der: dermis.

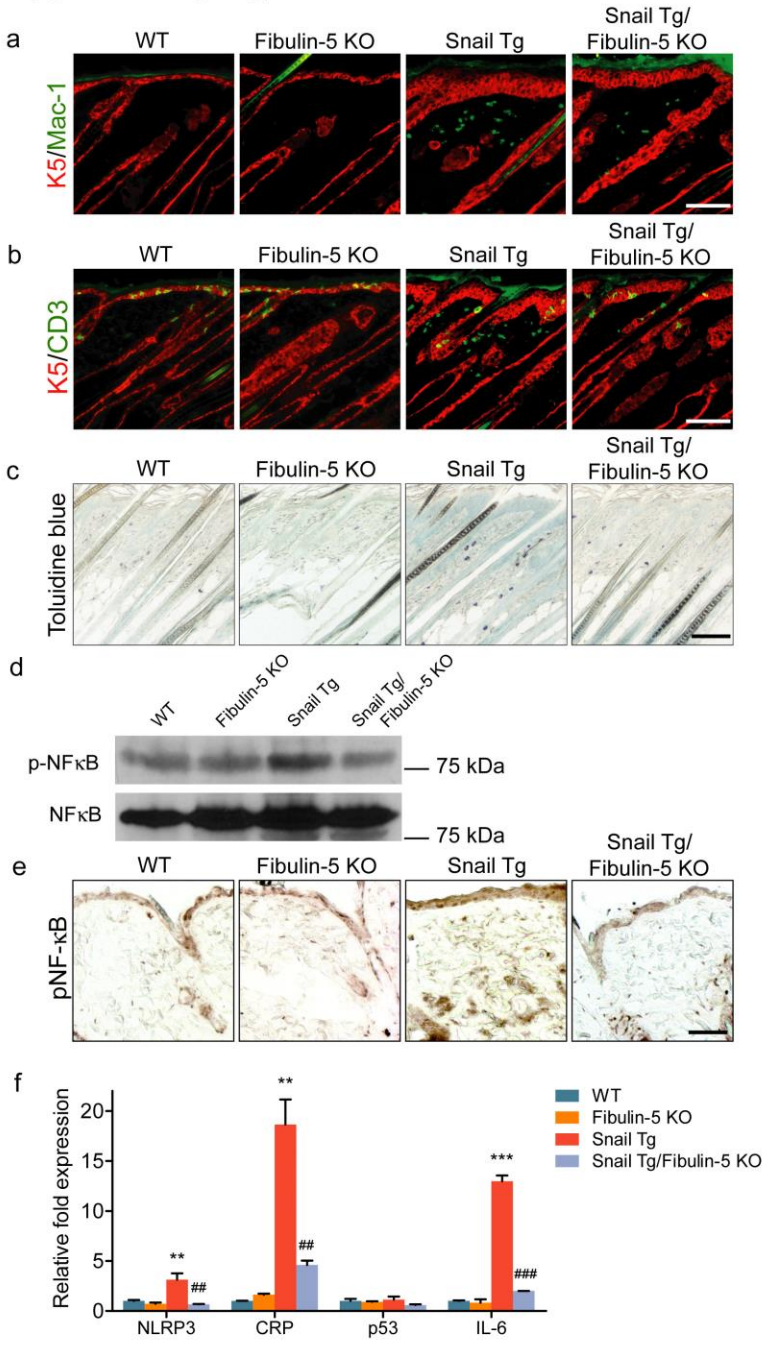

Supplementary Figure 2 Loss of fibulin-5 decreased inflammatory response in the Snail Tg skin. P9 skin sections from WT, Fibulin-5 KO, Snail Tg, Snail Tg/Fibulin-5 KO mice were stained with antibodies recognizing (a) macrophages (Mac-1, green), (b) T-cells (with the pan T-cell marker CD3, green) and keratin 5 (K5; red) and (c) mast cells using toluidine blue dye. (a-c) Bar, 50 μm. (d) Protein extracts were prepared from whole skin from P9 mice and probed

with an anti-phosphorylated NFκB (p-NFκB) or an anti-NFκB as a control. (e) Skin sections from 8-week-old WT, Fibulin-5 KO, Snail Tg, and Snail Tg/Fibulin-5 KO mice were subjected to immunohistochemical analyses with antibodies recognizing NFκB, a key regulator of proinflammatory genes. Bar, 50 μm. (f) Quantitative PCR of inflammation-related genes in WT (blue), Fibulin-5 KO (orange), Snail Tg (red), Snail Tg/Fibulin-5 KO (light blue) whole skin of P9 mice. Data represent mean ± SD of triplicate samples. $^{**}P < 0.01$ and $^{***}P < 0.001$ as compared with WT. $^{\#\#}P < 0.01$ and $^{\#\#\#}P < 0.001$ as compared with Snail Tg. Statistical analyses were performed with Student's t-test.

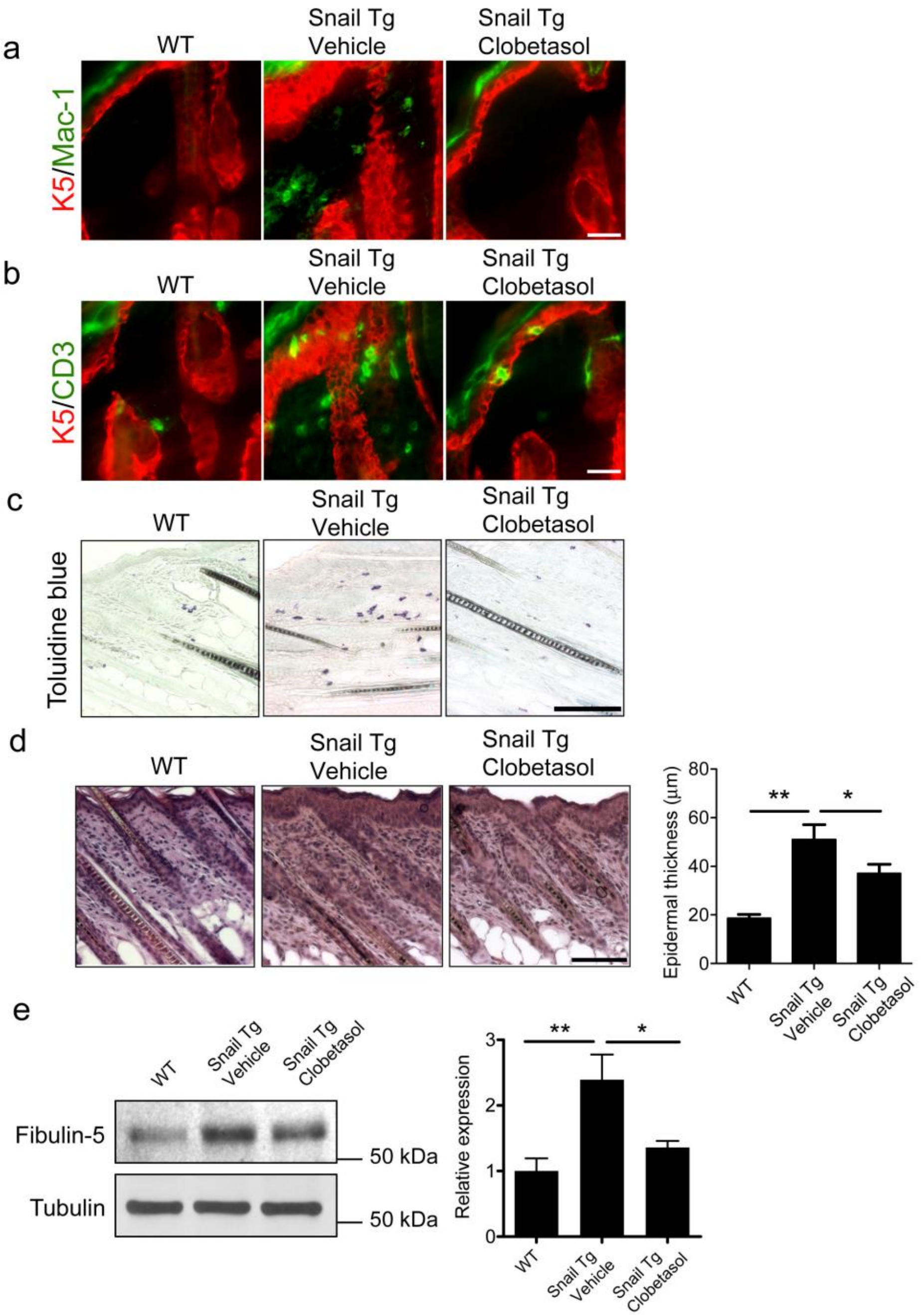

Supplementary Figure 3 Effect of immunosuppression on the fibrotic phenotype of the Snail transgenic skin. Vehicle control (Snail Tg Vehicle) or clobetasol (Snail Tg Clobetasol) was topically applied on newborn mice every other day until the age of 9. P9 skin sections were stained with with antibodies recognizing (a) macrophages (Mac-1, green), (b) T-cells (with the pan T-cell marker CD3, green) and keratin 5 (K5; red) and (c) mast cells using toluidine blue dye.

(a-c) Bar, 50 μm (d) Hematoxylin- and eosin-stained P9 skin sections. Bar, 100 μm. The right graph shows the comparison of epidermal thickness. Data represent mean ± SD of three samples. $^{*}P < 0.05$ and $^{**}P < 0.01$. Statistical analyses were performed by the unpaired Mann-Whitney *U* test. (e) Protein extracts were prepared from the P9 whole skin of WT and Snail Tg treated either control vehicle or clobetasol. Equal amounts of proteins were resolved by SDS-PAGE and subjected to Western blotting using either an anti-fibulin-5 antibody or an anti-tubulin as a control. The bar graph reflects quantitative analysis of relative fibulin-5 band intensities normalized by those for tubulin. Data represent mean ± SD of three samples. $^{*}P < 0.05$ and $^{**}P < 0.01$. Statistical analyses were performed with Student's t-test.

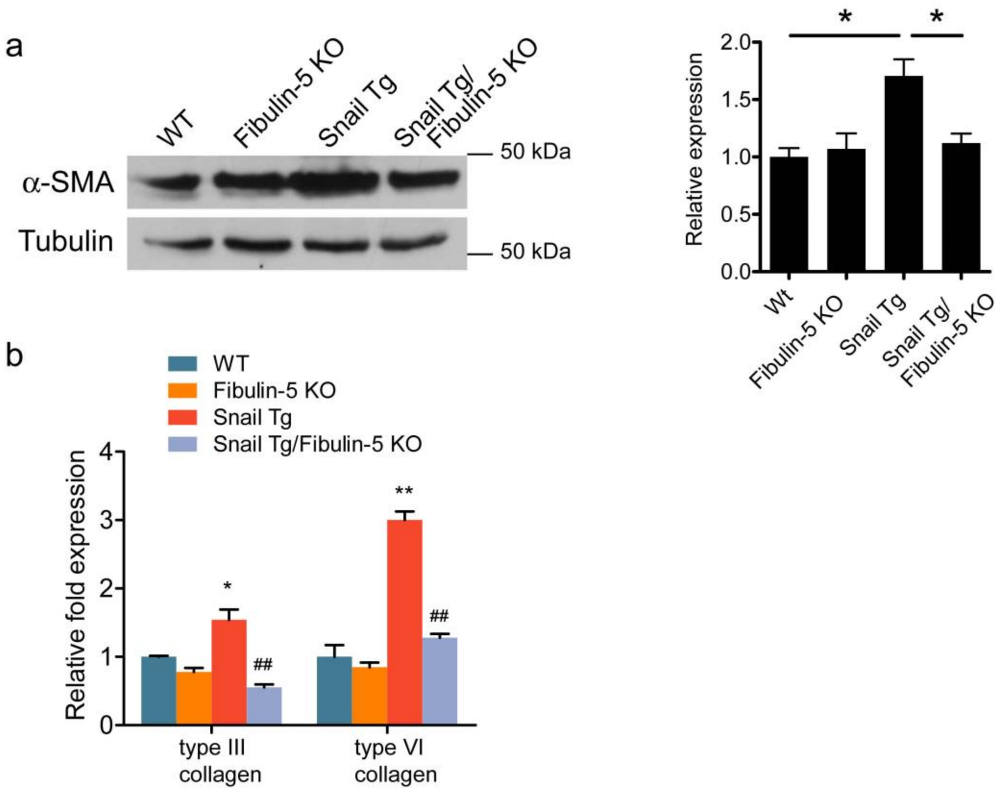

Supplementary Figure 4 A lowered level of α-smooth muscle actin in Snail Tg skin upon fibulin-5 depletion. (a) Protein extracts were prepared from whole skin from P9 mice. Equal amounts of proteins were resolved by SDS-PAGE and subjected to Western blotting using either an anti-α-smooth muscle actin (SMA), or an anti-tubulin as a control. The bar graph reflects quantitative analysis of relative α-SMA band intensities normalized by those for tubulin. Data represent mean ± SD of three samples. $^{*}P < 0.05$. (b) qPCR of collagen type III and VI genes in WT (blue), Fibulin-5 KO (orange), Snail Tg (red), Snail Tg/Fibulin-5 KO (light blue) whole skin of P9 mice. Data represent mean ± SD of triplicate samples. $^{*}P < 0.05$ and $^{**}P < 0.01$ as compared with WT. $^{\#\#}P < 0.01$ as compared with Snail Tg. Statistical analyses were performed with Student's t-test.

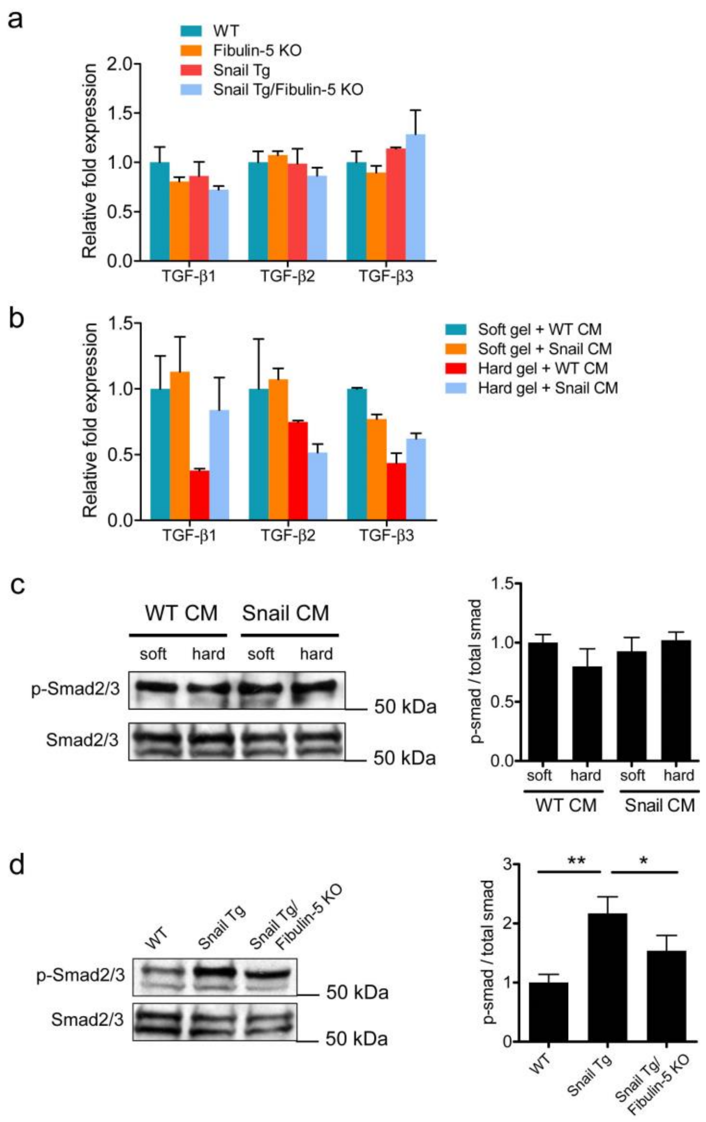

Supplementary Figure 5 TGF-β expression and signal transduction were not affected by ECM stiffness. (a) qPCR of TGF-β genes in the whole skin of P9 mice. Data represent mean ± SD of three samples. (b) Dermal fibroblasts were plated on collagen-coated polyacrylamide gels of varying stiffness (soft (2 kPa) and hard (5 kPa) gel). Cells were incubated with conditioned media from Snail Tg keratinocytes (Snail CM) or wild type keratinocytes (WT CM) for 18 hours. TGF-β gene expression in treated fibroblasts was determined by qPCR. (c) Dermal fibroblasts

were treated as in (b) and cell lysates were resolved by SDS-PAGE and subjected to Western blotting using either an anti-phospho-smad2/3 antibody or an anti-smad2/3 as a control. The bar graph reflects quantitative analysis of relative phospho-smad2/3 band intensities normalized by those for total smad2/3. Data represent mean ± SD of triplicate samples. (d) Protein extracts prepared from the P9 whole skin of WT, Snail Tg, and Snail Tg/Fibulin-5 KO were resolved and analyzed as in (c). Data represent mean ± SD of triplicate samples. $^{*}P < 0.05$ and $^{**}P < 0.01$. Statistical analyses were performed with Student's t-test.

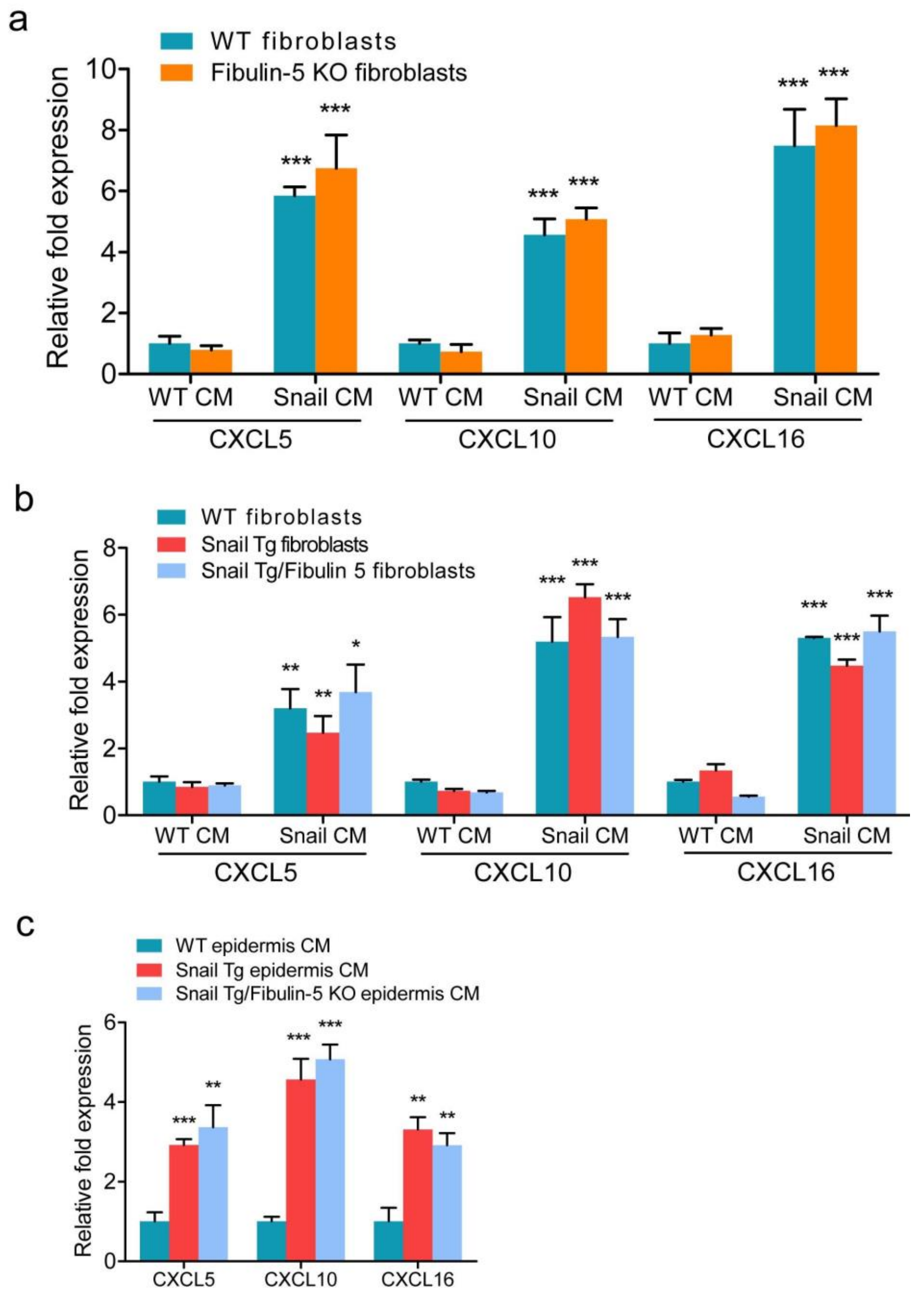

Supplementary Figure 6 Cell-intrinsic effect of Fibulin-5 in chemokine gene expression. (a) Dermal fibroblasts isolated from WT or Fibulin-5 KO skin were plated on collagen-coated 5 kPa polyacrylamide gels. Cells were incubated with conditioned media from Snail Tg keratinocytes (Snail CM) or wild type keratinocytes (WT CM) for 18 hours. Chemokine gene expression in

treated fibroblasts was determined by qPCR. Data represent mean ± SD of triplicate samples. $^{***}P < 0.001$ as compared with WT CM. (b) Dermal fibroblasts isolated from WT, Snail Tg, or Snail Tg/Fibulin-5 KO skin were treated as in (a) and chemokine gene expression in treated fibroblasts was determined by qPCR. Data represent mean ± SD of triplicate samples. $^{*}P < 0.05$, $^{**}P < 0.01$, and $^{***}P < 0.001$ as compared with WT CM. (c) Dermal fibroblasts were plated on collagen-coated 5 kPa polyacrylamide gels. Cells were incubated with media conditioned with epidermis isolated from WT, Snail Tg or Snail Tg/Fibulin-5 KO mouse skin for 18 hours. $^{**}P < 0.01$ and $^{***}P < 0.001$ as compared with WT epidermis CM. (a-c) Statistical analyses were performed with Student's t-test.

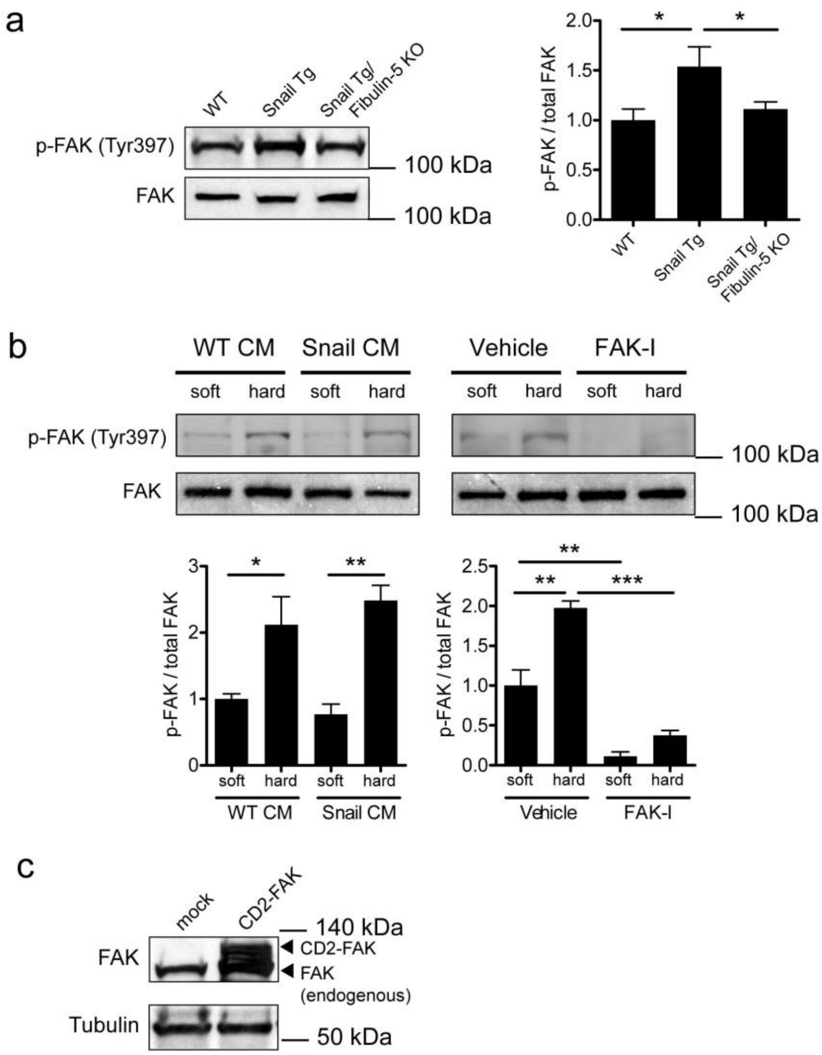

Supplementary Figure 7 Increased FAK phosphorylation in dermal fibroblasts on stiff matrices. (a) Protein extracts prepared from the P9 whole skin of WT, Snail Tg, and Snail Tg/Fibulin-5 KO were resolved by SDS-PAGE and subjected to Western blotting using either an anti-phospho-FAK antibody or an anti-FAK as a control. The bar graph reflects quantitative analysis of relative phospho-FAK band intensities normalized by those for total FAK. Data represent mean ± SD of triplicate samples. $^{*}P < 0.05$. (b) Dermal fibroblasts were plated on collagen-coated polyacrylamide gels of varying stiffness (soft (2 kPa) and hard (5 kPa) gel). Cells

were incubated with conditioned media from Snail Tg keratinocytes (Snail CM) or wild type keratinocytes (WT CM) for 18 hours. Cells were also treated with or without the presence of FAK inhibitor, PF 573228 at the concentration of 20 μM (FAK-I). Cell lysates were blotted and analyzed as in (a). Data represent mean ± SD of triplicate samples. $^{*}P < 0.05$, $^{**}P < 0.01$ and $^{***}P < 0.001$. (c) The ectopic expression of constitutively active form of FAK protein (CD2-FAK). Transfected cell lysates were blotted using either an anti-FAK antibody or anti-tubulin as a control. (a-b) Statistical analyses were performed with Student's t-test.

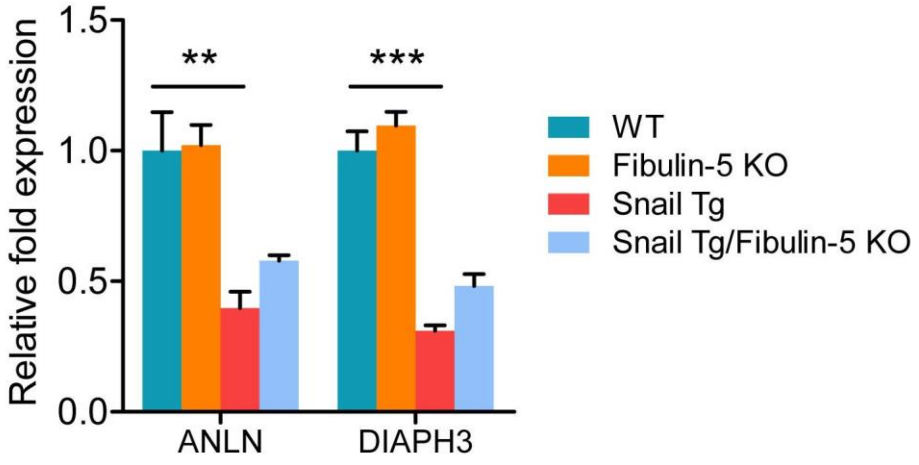

Supplementary Figure 8 Decreased YAP target gene expression in the Snail Tg skin. qPCR of YAP target genes, ANLN and DIAPH3 in the whole skin of P9 mice. Data represent mean ± SD of three samples. $^{**}P < 0.01$ and $^{***}P < 0.001$. Statistical analyses were performed with Student's t-test.

Supplementary Table 1 Systemic sclerosis sample information.

Biopsy number	Age	Sex	Disease duration	Modified Rodnan Skin Score
SSC 5	41	F	2 years	36
SSC 212	34	F	3 years	47
SSC 166	31	F	1.5 years	42
SSC 116	45	M	6 months	41
SSC 111	44	M	1.5 years	8
SSC 8	46	M	2 years	32
SSC 7	48	F	3 years	44
SSC 6	43	M	1 year	26
SSC 4	45	M	1 year	55
SSC 3	40	F	4 months	22
SSC 299	42	F	6 months	21
